# Epidemiology of *Leishmania* Carriers in Tan Chang County, Gansu Province, China

**DOI:** 10.3389/fcimb.2021.645944

**Published:** 2021-03-25

**Authors:** Shuai Han, Sheng-bang Chen, Zhang-hong Yang, Yu Feng, Wei-ping Wu

**Affiliations:** ^1^ National Institute of Parasitic Diseases, Chinese Center for Disease Control and Prevention, Shanghai, China; ^2^ Chinese Center for Tropical Diseases Research, Shanghai, China; ^3^ Key Laboratory of Parasite and Vector Biology, Ministry of Health, Shanghai, China; ^4^ WHO Collaborating Centre for Tropical Diseases, Shanghai, China; ^5^ National Center for International Research on Tropical Diseases, Ministry of Science and Technology, Shanghai, China; ^6^ Gansu Province Center for Disease Control and Prevention, Lanzhou, China; ^7^ Tan Chang County Center for Disease Control and Prevention, Longnan, China

**Keywords:** Kala-azar, *Leishmania*, host, nested PCR, rk39

## Abstract

**Background:**

Leishmaniasis is a regional infectious disease caused by the bite of *Leishmania*-carrying sandflies. The clinical symptoms include prolonged fever, spleen enlargement, anemia, emaciation, leukopenia, and increased serum globulin levels. If not appropriately treated, patients may die of complications caused by leishmaniasis within 1–2 years after the onset of the illness. Therefore, further investigation of the mechanisms of infection by this pathogen is required. Here, an epidemiological study of *Leishmania* carriers was conducted. The potential mechanism of infection through domestic animals as carriers of the parasite was investigated to identify potential reservoir hosts for *Leishmania.*

**Methods:**

The rK-39 strip test was performed on blood samples from previously infected patients. Blood samples were collected from the patients and their families. The blood, liver, spleen, and diaphragm muscle samples were collected from livestock. To perform nested polymerase chain reaction (PCR), DNA was extracted and the internal transcribed spacer sequence was used. The amplified products were then subjected to restriction fragment length polymorphism and phylogenetic analyses.

**Results:**

Among previously infected patients, 40% (12/30) showed positive results in the rK-39 strip test. The nested PCR positive rates for previously infected patients/relatives and livestock samples were 86% (77/90) and 80% (8/10), respectively. Moreover, the phylogenetic analysis showed that the pathogen was *Leishmania infantum*. Dogs, patients, and domesticated animals carrying *Leishmania* were found to be a potential source of infection for leishmaniasis.

**Conclusions:**

The results of this study provide a basis for developing disease prevention and control strategies for leishmaniasis.

## Introduction

Kala-azar, also known as visceral leishmaniasis (VL), is a regional infectious disease caused by the bite of *Leishmania*-carrying sandflies. Clinical symptoms include prolonged irregular fever, spleen enlargement, anemia, emaciation, leukopenia, and increased serum globulin levels. If not treated appropriately, most patients would die of VL-related complications within 1–2 years after the onset of illness (Desjeux, 1996; Boelaert et al., 2000; Alvar et al., 2006). The annual incidence of VL is around 200,000–400,000 cases globally; of these cases, over 90% are distributed in six countries including India, Bangladesh, Sudan, South Sudan, Ethiopia, and Brazil (Guerin et al., 2002; de Vries et al., 2015). Kala-azar is a disease that can be easily overlooked and whose severity is often underestimated (Singh et al., 2006; Mosleh et al., 2008; Zijlstra, 2016). Therefore, a trend of kala-azar outbreaks has been noticed in recent years (Wang et al., 2010).

In China, 650 endemic VL cases were reported in the 1950s. The disease was eliminated from the major epidemic areas of the country by the end of the 1950s. Currently, there are about 200 to 500 VL cases in China each year and these cases are sparsely distributed in different rural areas. The endemic areas have been classified into three types according to geography, pathogen, and medium: plain, mountainous, and desert areas (Lu et al., 1994; Wang et al., 2012). The Longnan Region in the Gansu Province is one of the current endemic areas for leishmaniasis in China and the pathogen found within this region is *Leishmania infantum* (Wang et al., 2000; Li and Gao, 2001; Yu et al., 2015; Zheng et al., 2017). The mountainous type of VL is endemic to Tan Chang County, situated in the northwestern part of the Longnan Region, Gansu Province. Tan Chang County is one of the primary locations of VL incidents (Wei et al., 2008) and the patient population within this county primarily consists of children. According to the Infectious Disease Report System, 87 VL cases have been reported in Tan Chang County since 2006, with an increasing number of patients identified each year. Although these VL cases are often sparsely distributed and most patients reside in small villages such as Shawan, Xinzhai, Guanting, and Lianghekou, the disease has continued to spread without intermission, and the number of patients and the size of the affected area are increasing each year.

Previously, dogs were thought to be the major animal source of infection for kala-azar (Smeyne et al., 1995; Dantas-Torres, 2007). However, studies on *Leishmania*-infected dogs suggested that most carrier dogs do not show any symptoms of the disease. Rather, they appear to be asymptomatic or not show any signs of infection (Boelaert et al., 2000; Caldas et al., 2002; Wang et al., 2007). Therefore, asymptomatic animals carrying the parasite may play a substantial role in transmitting the disease (Molina et al., 1994; Bern et al., 2007; Ferreira et al., 2009; Fu et al., 2009; Topno et al., 2010; Moreno et al., 2014; Gao et al., 2015; Esteva et al., 2017). Accurate estimation and confirmation of the number of leishmaniasis-affected asymptomatic dogs are critical for controlling the disease (Ashford et al., 1998; Zhang et al., 2008). Some researchers have proposed that the areas where the disease is endemic may be the ones from which the disease originated (Xiong and Jin, 2003). Given that local residents regularly take dogs with them into the mountains for hunting or guarding crops, *Leishmania* parasites can be regularly found in dogs that roam in the mountains. An analysis of the potential animal reservoirs suggests that the source of infection may originate from certain wild animals that reside within the mountains; hence, the possibility of additional animal hosts in the wilderness cannot be excluded (Jin et al., 2007; Souza et al., 2014). Because of local geographical features, houses generally consist of two stories, with the ground level designated for livestock rearing and the second level designated for living. As a result, villagers regularly come into contact with domesticated animals on a daily basis, which makes these domesticated animals another potential host for *Leishmania* (Bhattarai et al., 2010; Singh et al., 2013).

In recent years, as the number of domesticated dogs has increased along with population mobility, leishmaniasis has shown a tendency to spread again. Therefore, to understand the current status of the leishmaniasis epidemic in Tan Chang County and to identify potential hosts for *Leishmania*, a scientific investigation of the distribution of hosts and the associated infection status was conducted in Tan Chang County in December 2014.

## Materials and Methods

### Collection of Blood Samples From Patients and Their Relatives, and of Blood and Tissue Samples From Livestock

To examine the kala-azar awareness and the patients’ treatment conditions, patients identified by local records were provided with a questionnaire where they were asked about their living habits and environment. All questionnaires were answered anonymously. Written informed consent was obtained from all adult participants that answered the questionnaire and from whom blood samples were collected. In the case of child participants, written informed consent was obtained from the parents or guardians for questionnaire participation and blood sample collection.

**Figure 1:**
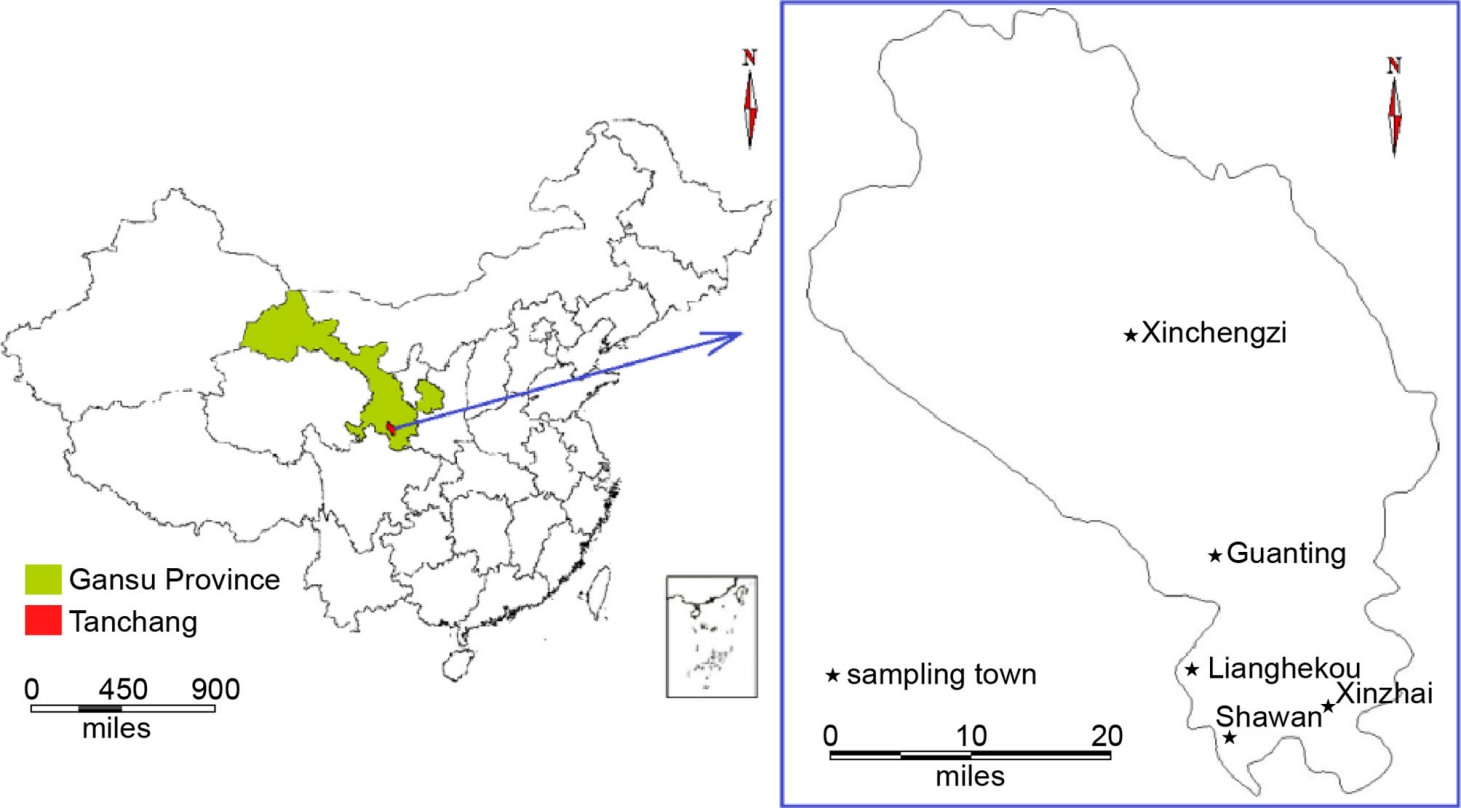
Distribution of selected villages for blood sample collection.

In total, 29 households across five villages were included in this study based on the national notifiable disease report system database (**Table 1**; **Figure 1**). Blood samples (3 mL) were collected from patients and their relatives living in the same house (for a total of 90 samples) using 6-mL EDTA blood collection tubes (BD Vacutainer; Becton Dickinson, Franklin Lakes, NJ, USA). Tubes were kept at 4°C until analysis.

**Table 1:** Distribution of Visceral Leishmaniasis Cases in the Tan Chang County (up to 2014).

Village	Population (from 5^th^ National Census)	Number of patients with visceral leishmaniasis									
		2006	2007	2008	2009	2010	2011	2012	2013	2014	Total
Shawan	24778	2	3		4	5	4	1	2	5	26
Guanting	6713				2		2			2	6
Lianghekou	12361				1	1	3	2	4	2	13
Xinzhai	12425			6	3	5	7	4	1	4	30
Xinchengzi	6129						2		1		3
Nanyang	13486						2			1	3
Lichuan	11816					1					1
Hadapu	13118							1			1
Shizi	5905							1			1
Chela	11721									1	1
Unknown										2	2
Total		2	3	6	10	12	20	9	8	17	87

The 29 households containing previously infected patients typically reared livestock and dogs, with an average of 1.3 livestock per household (38/29) and 0.7 dogs per household (21/29), which was significantly higher than the numbers in other households in these five villages.

Local slaughterhouses were also selected for blood sample collection with the approval and supervision of the Animal Husbandry and Veterinary Department of Tan Chang County. The blood (3 mL), liver, spleen, and diaphragm muscle tissue samples (5 g) were collected from nine livestock pigs. Specifically, liver samples from three sheep and two pigs, blood samples from two pigs, spleen sample from one pig, and diaphragm muscle sample from one pig were collected. Notably, this study did not involve any animal raising or animal sacrifice, and the collections were scheduled in advance and did not change the routine procedures used at the slaughterhouses.

### rK39 Strip Test

Whole blood (20 µL) was dripped onto the sample application site of an rK39 strip (InBios, Seattle, WA, USA) on a sample application pad. Next, 1–2 drops of PBS were added, and the strip was laid flat for 5–10 min. All strips were then visually examined.

### DNA Extraction

DNA was extracted using an SE Blood DNA Kit (Omega Bio-Tek, Norcross, GA, USA) following the manufacturer’s instructions. Each DNA extraction required 300 μL of whole blood. The obtained DNA was kept at −20°C until use.

### Nested PCR

DNA from blood samples was extracted using the SE Blood DNA Kit (Omega Bio-Tek) according to the protocol in the instructions manual (Wang et al., 2006). The conserved internal transcribed spacer (*ITS*)-1 gene between the genes encoding for *SSU* rRNA and 5.8S rRNA was used as the target gene in this analysis (Schonian et al., 2003). Nested PCR was performed as described by Ferreira et al. using the reported amplification protocol and primers (Katakura et al., 1998; Carvalho Ferreira et al., 2014). The primers were synthesized by Invitrogen Trading Co., Ltd, Shanghai, China. In the first round of PCR, the reaction volume was 50 μL, containing 1 μL of DNA template, 25 μL of MAX PCR Master Mix (Takara Biotechnology Co., Ltd, Dalian, China), and 1 μL of each primer; the concentration of primers used in each reaction was 10 μM. The primers were as follows: forward, 5′-CTGGATCATTTTCCGATG-3′ and reverse, 5′-TGATACCACTTATCGCACTT-3′. For every reaction, a positive control (*L. infantum* MHOM/CN/08/JS-1 DNA) and negative controls were used. Conditions for the nested PCR were as follows: initial denaturation at 94°C for 5 min; 30 cycles of denaturation at 94°C for 30 s, annealing at 53°C for 30 s, and extension at 72°C for 30 s; and a final extension at 72°C for 5 min. The amplification products from the first round of PCR were then diluted 1:40 and used as a template for the second round of PCR performed in a reaction volume of 25 μL, including 12.5 μL of MAX PCR Master Mix and 1 μL of each primer (10 μM; sequences: forward, 5′-CATTTTCCGATGATTACACC-3′ and reverse, 5′-CGTTCTTCAACGAAATAGG-3′). The reaction conditions were the same as those in the first round of PCR. The expected size of the PCR product was approximately 280–330 bp. The product from the second round of PCR (5 µL) was loaded onto a 1.5% agarose gel and subjected to electrophoresis. The PCR products were then visualized using a gel imager.

### Restriction Fragment Length Polymorphism Analysis

Positive nested PCR products were digested using the restriction endonuclease *Hae*III (1 μL, 50 ng/μL; TaKaRa Biotechnology Co., Ltd.) in a 20-μL reaction containing 2 μL of 10× M buffer and 1 μg or less of DNA. The mixture was incubated at 37°C in a water bath for 1 h; 5 µL of the digested product was subjected to electrophoresis on 1.5% agarose gels, and RFLP analysis was then performed. If the RFLP analysis of different nested PCR products showed the exact same RFLP pattern, the products were categorized as having originated from the same visceral *Leishmania* species. Different positive nested PCR products were sent to Life Technologies Co. Ltd., Shanghai, China for sequencing.

### Phylogenetic Analysis and Cladogram Construction

The sequences were edited using the DNAstar software (DNASTAR Inc., Madison, WI, USA). BLAST (National Center of Biotechnology Information, NCBI) was used to identify published sequences homologous to the target sequence and to determine *Leishmania* species. The sequence alignments and phylogenetic analyses of the aligned sequences were performed using ClustalW and the neighbor-joining method, respectively, in the MEGA 5.0 software package (DNASTAR Inc.).

## Results

### rK39 Strip Test Results

Among previously infected patients, 40% (12/30) showed positive results for the rK-39 strip test (**Table 2**). The rK39 strip test was not performed on relatives of previously infected patients.

**Table 2:** Test results of patients previously infected with visceral leishmaniasis and their relatives.

Sample population	rK39 test			Nested PCR test		
	Number	Positive number	Positive rate	Number	Positive number	Positive rate
Patient	30	12	40.00%	30	28	93.33%
Patient relatives	N/A	N/A	N/A	60	49	81.67%
Total	30	12	40.00%	90	77	85.56%
χ^2^						2.2028
p-value						0.1377

N/A, not available.

### Assessment of *L. infantum* Infection Rates Using Nested PCR Analysis

**Figure 2:**
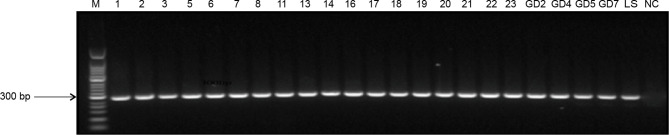
Nested PCR amplification of *ITS* genes from *Leishmania* species. M, 50-bp DNA marker; 1–23, DNA extracted from patient blood samples and samples from their family members; GD2–GD7, DNA extracted from animal tissues of pigs; LS, positive control; NC, negative control.

As expected, 285-bp bands (**Figure 2**) indicated the target gene amplified by nested PCR, and no bands of other sizes were present. Data collected from previously infected patients and relatives indicated that the infection rate by *L. infantum* was 86% (77/90). The infection rate in animal blood and tissue samples was 80% (8/10). Liver samples from a sheep and a pig were negative for the infection. Thus, these data suggested that previously infected patients and their relatives (even without VL symptoms), and domestic livestock may serve as potential hosts for *L. infantum* parasites.

### RFLP Analysis

**Figure 3:**
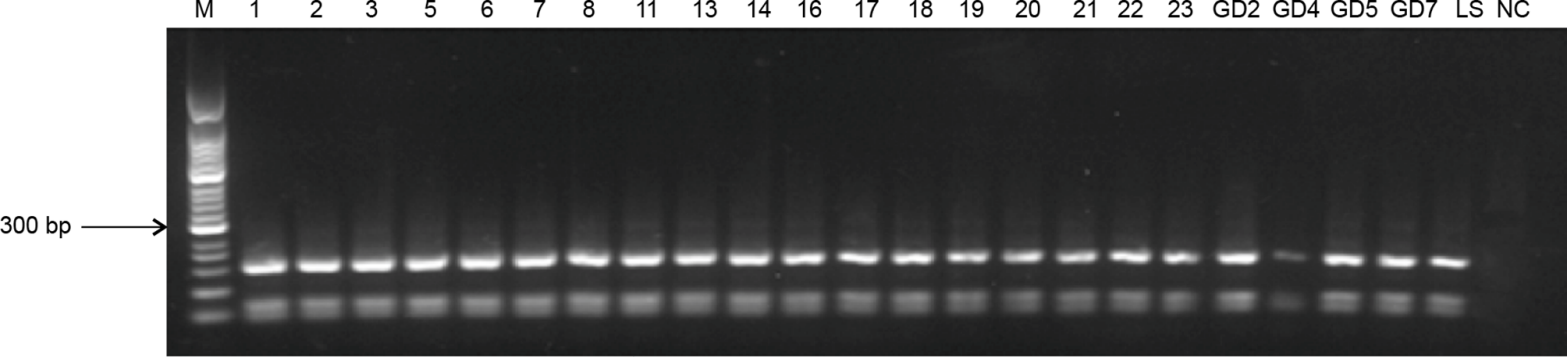
RFLP profiles of *ITS* genes amplified from *Leishmania* digested with *Hae*III. M, 50-bp marker; 1–23, blood samples from infected patients and their families; GD2–GD7, blood and tissue samples from domesticated pigs; LS, positive control; NC, negative control.

The RFLP analysis indicated exact matches for all PCR products following restriction digestion (**Figure 3**). The fragments had a length of 161, 69, and 55 bp, suggesting that all the bands originated from the same *Leishmania* species.

### Phylogenetic Analysis

**Figure 4:**
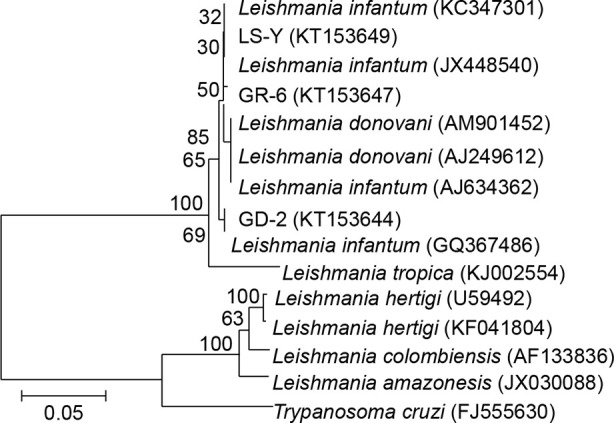
Phylogenetic analysis of *ITS* genes in *Leishmania*. LS-Y (MHOM/CN/08/JS-1), positive control; GR-6, blood from kala-azar patient; GD-2, animal tissue from pigs.

The GR-6, GD-2, and LS-Y (MHOM/CN/08/JS-1) target sequences all belonged to the same group on the phylogenetic tree, indicating that local villagers and domesticated livestock were infected by the same type of *L. infantum*. Additionally, the sequences from GR-6 and GD-2 belonged to the same cluster, offering further evidence that the infection of people and domesticated livestock was related (**Figure 4**).

## Discussion

Herein, a case study of *Leishmania* carriers and the potential infection route of the parasites through domestic animals was performed to investigate the potential reservoir hosts for the parasites. Our data demonstrated that domesticated livestock may serve as carriers for *Leishmania* and provide insights into the origins of the infection in different families and individuals from Tan Chang County, China.

According to Xiong and Jin, (2003) the mountainous type of leishmaniasis and its local spread pattern in this region suggested that this type represented the natural cause of the epidemic in humans and dogs. It was believed that the parasite was passed to dogs from natural wildlife and subsequently spread from dogs to humans. When the parasite was transmitted from one host to another or between different hosts, the lifecycle of the sandfly played a pivotal role (Ashford et al., 1998), allowing parasites to spread among local wildlife and maintaining the presence of *Leishmania* in its natural habitat, while at the same time transmitting it to humans and dogs and causing visceral leishmaniasis.

The villages in Tan Chang County are fairly spread out. Moreover, the unique characteristics of the residential houses create perfect conditions for sandflies to thrive and spread (Li et al., 2004). The results of the rK39 strip test for previously infected patients showed a high positive rate, suggesting that they still produced *L. infantum*-related antibodies (Molinet et al., 2013; Regina-Silva et al., 2014). Locally, the numbers of domesticated sheep and pigs are fairly high, and with the improvement in the quality of life, the number of pet dogs has also increased. Patients previously infected with *L. infantum* had on average 1.3 (38/29) livestock animals and 0.7 (21/29) dogs per household, and these numbers are significantly higher than the local average. Studies of the transmission dynamics of visceral *Leishmania* by Stauch et al. (2011) in India indicated that asymptomatic carriers are the main factor in the spread of the disease (Bern et al., 2007; Fu et al., 2009; Stauch et al., 2011; Tiwary et al., 2017). Furthermore, we cannot exclude the possibility that asymptomatic domesticated *Leishmania*-carrying animals may also be a major factor affecting the maintenance of the *Leishmania* epidemic in this endemic region. As the lifestyle of local villagers includes frequent contact with and close proximity to domesticated animals, the risk of parasite transmission is greatly increased.

The standard test for leishmaniasis is the rK39 strip test (Maia et al., 2012; Srividya et al., 2012; Georgiadou et al., 2015). However, this test can only be used on symptomatic patients and cannot detect asymptomatic carriers (Burns et al., 1993; Guan et al., 2001; Matlashewski et al., 2013; Molinet et al., 2013; Regina-Silva et al., 2014). Thus, an alternative method for the detection of asymptomatic carriers is needed. The *ITS-1* sequence located between the genes encoding *SSU* rRNA and 5.8S rRNA is not transcribed into RNA. During evolution, this region has accumulated many mutations, and the degree of variation in ITS-1 sequence can reflect the genetic relationships during biological evolution. Therefore, sequence analysis of *ITS-1* is widely used in evolutionary research for the classification of various biological systems (Villalba and Ramirez, 1982; Villalba et al., 1985). Leite et al. (2010) used a combination of *ITS-1* nested PCR and Kinetoplast DNA PCR in asymptomatic dogs infected with *L. infantum* to assess the efficacy of the hybrid test. This analysis showed that *ITS-1* nested PCR analysis of conjunctiva swab samples exhibited high sensitivity (up to 83.3%), whereas that of samples from dog blood displayed a relatively lower sensitivity (56.7%). The sensitivity of both of these tests is much higher than that of the kDNA PCR hybrid test (13.3%). Pilatti et al. (2009) also performed *ITS-1* nested PCR in symptomatic dogs and observed a sensitivity of 73.9%. Thus, these data showed that *ITS-1* nested PCR displays a fairly high sensitivity for detection of *L. infantum* in symptomatic and asymptomatic dogs (Di Muccio et al., 2012; Ajaoud et al., 2013).

In this study, rK39 strip tests showed 40.00% positivity (12/30) in blood samples from patients previously infected with VL, which indicates that these previously infected patients still presented symptoms of kala-azar. Nested PCR on DNA extracted from these blood samples was also used to assess the *L. infantum* infection status in previously infected patients and their relatives. Our results showed that the infection rate was 86% (77/90), suggesting that local residents may have a widespread history of sandfly bites and are asymptomatic carriers at high risk of developing the disease. Moreover, the infection rate in animal blood and tissue samples was 80% (8/10), and our phylogenetic analysis showed that GR-6, GD-2, and LS-Y (MHOM/CN/08/JS-1) belonged to the same cluster, indicating that local villagers and domesticated livestock were both infected with the same strain of *L. infantum*. Additionally, GR-6 and GD-2 belonged to the same cluster, suggesting that the *Leishmania* infections in humans and domestic animals were linked.

Given that the time of sampling did not coincide with the sandfly transmission season, it was suspected that symptom-free patients and domesticated livestock might also be potential hosts for *Leishmania*. Indeed, it was found that not only dogs, but also other livestock, could be animal *Leishmania* hosts. Thus, this study has expanded the current understanding of the types of *Leishmania* hosts and may facilitate the implementation of targeted preventive measures to effectively control the disease. However, because of the small sample size and long storage time of the samples used in our study, we cannot yet confirm the presence of *Leishmania* in all tested hosts and patient groups, and further studies are required.

## Conclusions

This report provides insights into the potential mechanism of infection by *Leishmania* and identifies potential factors promoting the spread of leishmaniasis, which is an overlooked disease with no current prevention strategies. The treatment of patients with leishmaniasis is very challenging due to the lack of knowledge about the disease transmission and the asymptomatic nature of the pathogen. rK39 strip test results, nested PCR, and phylogenetic analyses demonstrated that the patients and domesticated livestock were infected by the same type of *L. infantum*. Thus, dogs, patients, and livestock carrying *Leishmania* could be potential sources of infection. Therefore, implementation of directed control and epidemic monitoring might help in preventing leishmaniasis outbreaks in endemic regions.

## Data Availability Statement

The datasets presented in this study can be found in online repositories. The names of the repository/repositories and accession number(s) can be found below: https://www.ncbi.nlm.nih.gov/genbank/, KT153649 https://www.ncbi.nlm.nih.gov/genbank/, KT153647 https://www.ncbi.nlm.nih.gov/genbank/, KT153644.

## Ethics Statement

The studies involving human participants were reviewed and approved by National Institute of Parasitic Diseases, Chinese Center for Disease Control and Prevention. Written informed consent to participate in this study was provided by the participants’ legal guardian/next of kin.

## Author Contributions

SH, W-pW, and YF conceived and designed the experiments. SH, W-pW, S-bC, and Z-hY collected the samples and performed the experiments. SH and W-pW were the major contributors in purchasing reagents and materials, data collection and analysis, and writing the manuscript. All authors contributed to the article and approved the submitted version.

## Funding

This work was supported by the Research Project of Shanghai Municipal Health Commission(No.202040052) and National Key Technology Research and Development Program (No. 2014BAI13B05).

## Conflict of Interest

The authors declare that the research was conducted in the absence of any commercial or financial relationships that could be construed as a potential conflict of interest.

## Abbreviations

EDTA, Ethylenediaminetetraacetic acid; kDNA, kinetoplast DNA; PBS, phosphate-buffered saline; PCR, Polymerase chain reaction; RFLP, restriction fragment length polymorphism; rRNA, ribosomal RNA; VL, Visceral leishmaniasis.
